# COVID-19 vaccine intentions and attitudes in Black American emerging adults with asthma

**DOI:** 10.1186/s12889-024-18843-w

**Published:** 2024-05-20

**Authors:** Amy Lee Hall, Pranati Movva, Rhonda Dailey, Wanda Gibson-Scipio, Alan P. Baptist, Karen Kolmodin MacDonell

**Affiliations:** 1 College of Nursing, Wayne State University, Detroit, MI USA; 2 Department of Behavioral Sciences and Social Medicine, Center for Translational Behavioral Science, Florida State University College of Medicine, Tallahassee, FL USA; 3 Michigan State University College of Osteopathic Medicine, Lansing, MI USA; 4 School of Medicine, Wayne State University, Detroit, MI United States of America; 5 Henry Ford Health, Division of Allergy and Immunology, Detroit, MI USA

**Keywords:** Asthma, Black adult, Emerging adults, COVID-19 vaccine

## Abstract

**Background:**

Emerging adults (aged 18–29) are less likely to receive the COVID-19 vaccine than any other adult age group. Black Americans are less likely than non-Hispanic white Americans to be fully vaccinated against COVID-19. This study explored factors which affect vaccine intention and attitudes in Black American emerging adults with asthma.

**Methods:**

Participants were recruited from an NHLBI-funded clinical trial to improve asthma control. Fifty-nine Black American emerging adults completed a Qualtrics survey that assessed asthma control, intention to vaccinate, and factors which may affect the decision to vaccinate. Twenty-five participants also completed a semi-structured interview via Zoom. Bivariate correlations and descriptive statistics, including Chi Square analyses, were run using SPSS. Interview thematic analyses were conducted via QDA Miner.

**Results:**

Of the 59 Black American emerging adults with asthma who completed surveys, 32.2% responded that they were highly unlikely to receive the COVID-19 vaccine, while 50.8% responded that they were highly likely to receive it. Increased asthma control was significantly correlated with a higher likelihood to discuss the COVID-19 vaccine with their healthcare provider (ρ = 0.339, α = 0.011). Concerns about immediate (ρ= -0.261, α = 0.050) and long-term (ρ= -0.280, α = 0.035) side effects were inversely correlated with intention to vaccinate. Only 17% of the participants who were unemployed stated that they were highly likely to receive the vaccines compared to 65% of the participants who were employed; however, interview participants who were unemployed stated not needing the vaccine because they were protecting themselves by social distancing. When deciding whether to receive the vaccine, safety, efficacy, and immediate side effects were the top three factors for 91%, 54%, and 49% of the participants, respectively. Beliefs about the vaccines’ safety and efficacy, information gathering, personal factors, and societal factors emerged as important themes from the interviews.

**Conclusion:**

Only half of the surveyed Black American emerging adults with asthma were highly likely to receive the COVID-19 vaccine. Safety and efficacy were important for the majority of the participants, regardless of vaccine intention. Greater asthma control, but not access to asthma-related healthcare, was correlated with intention to discuss the vaccine with their healthcare provider.

**Supplementary Information:**

The online version contains supplementary material available at 10.1186/s12889-024-18843-w.

## Background

Three years into the global pandemic, vaccines remain an important tool to prevent the morbidity and mortality from COVID-19 [1]. Black American emerging adults with asthma, aged 18–29, are a priority population for COVID-19 vaccination. Although emerging adults are the least likely age group to have severe outcomes from COVID-19, they are the most likely to contract COVID-19, account for the most cumulative cases of COVID-19 [2], and drive community-wide infections [3]. Black American emerging adults with uncontrolled or severe asthma may themselves be at an increased risk for severe outcomes from COVID-19 infection as Black Americans are more than twice as likely to be hospitalized and 1.6 times as likely to die from COVID-19 than age-matched non-Hispanic Whites [4]. Additionally, severe and/or uncontrolled asthma requiring oral steroids or hospitalizations within the last two years has been shown to increase the risk for COVID-19 hospitalization [5, 6], thereby increasing the vulnerability of Black American emerging adults with asthma to COVID-19.

Black American emerging adults with asthma were at an increased risk for adverse health outcomes even before the onset of COVID-19. Emerging adulthood, ages 18–29 years old, is the transition from adolescence to adulthood, and includes developmental characteristics, such as increased risk-taking and independence and decreased parental support [7] that may contribute to adverse health outcomes. Emerging adults with chronic health conditions, such as asthma, are particularly vulnerable [8, 9]. As they age, emerging adults must transition from familiar pediatric healthcare settings into adult settings, often without appropriate transition planning or preparation [10, 11]. Additionally, older emerging adults in the United States may lose health insurance coverage as they age out of their parents’ health insurance coverage [12], and indeed, emerging adults have the lowest medical insurance coverage of any age group [13] and the highest utilization of the emergency department for medical care [14, 15]. Minority emerging adults experience this transition differently than their non-Hispanic White peers [16–18] and have been shown to be at an increased risk for negative physical and mental health outcomes [19]. While little is known about COVID-19 outcomes in Black American emerging adults with asthma, previous research has shown that COVID-19 exacerbated, rather than alleviated, existing health inequities [20].

COVID-19 vaccines are recommended for everyone over the age of 6 months, especially those with a chronic condition, and are an important tool to address COVID-19 related health inequities as they decrease the risk for hospitalization and death [1, 2, 21]. Data from the CDC, however, suggests both age- and race-based differences in vaccine administration rates. Less than 67% of emerging adults aged 18–24 reported receiving the primary series of the COVID-19 vaccine and only 7.4% reported receiving the recommended bivalent booster [22]. In contrast, 94% of adults aged 65 years and over reported receiving the primary series with 43.3% receiving the recommended bivalent booster. Vaccine administration data, although missing race and ethnicity data for a quarter of the population, suggests that Black Americans of all ages have been less likely to receive both the primary series (45% vs. 51.9%) and the bivalent booster (9.5% vs. 16.7%) than non-Hispanic Whites. Survey data suggests that Black Americans report being slightly more likely to receive the primary series (85.1% vs. 84.3%), but less likely to receive the bivalent booster (29.3% vs. 37.5%) than non-Hispanic Whites.

The World Health Organization has listed vaccine hesitancy as one of the top ten threats to global health [23]. Vaccine hesitancy, the refusal or delay in receiving recommended and available vaccines, has led to a resurgence of vaccine-preventable diseases, such as measles and polio. Rates of vaccine hesitancy vary by vaccine type, age, and race. For example, in the United States, Black Americans and emerging adults are less likely to receive the influenza vaccine than any other race or age group, respectively [24].

A useful framework to study vaccine hesitancy and intention is secondary risk theory, an extension of protection motivation theory (PMT) [25]. PMT posits that when individuals are faced with a risk, they will be more likely to perform recommended behaviors to reduce that risk when they: feel vulnerable to the risk, perceive the risk as severe, and feel that the recommended behavior is efficacious against the risk. Secondary risk theory extends PMT by stating that individuals also consider the risk of the recommended behavior itself. Indeed, concerns about the COVID-19 vaccine’s safety and side effects (secondary risks) emerged as the most common predictors of vaccine hesitancy in the general population in multiple systematic reviews [26–31]. Other predictors of vaccine hesitancy included individuals’ perceived risk from COVID-19 [27, 29, 31] and concerns about the vaccine’s efficacy [26, 32]. Some of the demographic predictors that have been associated with vaccine hesitancy, which may reflect perceived risk, perceived vulnerability, or perceived secondary risk, include identification as a member of a racial or ethnic minority [29, 33, 34], younger age [33–35], lower socioeconomic status [33], female gender [28, 29, 34], and unemployment [28].

While little is known about COVID-19 vaccine intentions in persons with asthma, COVID-19 vaccine acceptance in persons with other chronic conditions is related to concerns about the vaccine’s safety, side effects, and efficacy, as well as perceived risk of severe outcomes from COVID-19 [36–39]. As in the general population, younger age and identifying as a minority are related to lower vaccine intention in adults with chronic conditions [37, 39]. The decision to receive the COVID-19 vaccine may be more complicated for emerging adults with chronic disease than for older adults. On the one hand, emerging adults as an age group are at a lower risk for adverse health outcomes from COVID-19 than other adult age groups [2, 40]; however, having a chronic condition increases the risk for adverse COVID-19 outcomes [5]. The decision may be more complicated for minority emerging adults with chronic conditions due to race-related health inequities [4, 41]. For example, minority adults with asthma were more likely to live in communities with a high number of COVID-19 cases and report difficulty obtaining asthma medications than non-Hispanic Whites [42].

While emerging adults, including minority emerging adults, in general, report concerns about the COVID-19 vaccine’s safety, efficacy, and side effects, as well as a mistrust of the vaccine [43–46], little is known about vaccine intention in minority emerging adults with chronic conditions. To our knowledge, there have been no studies which explore vaccine intentions in Black American emerging adults with asthma or the relationship between vaccine intentions and asthma control.

The aims of this parallel convergent mixed methods study were to explore COVID-19 vaccine intentions, attitudes, and beliefs in Black emerging adults with asthma during the COVID-19 pandemic using secondary risk theory as a framework and to explore how asthma control was related to vaccine intentions. Quantitative data were collected and measured using surveys which assessed asthma control, demographic variables, beliefs about COVID-19, and beliefs and intentions related to the COVID-19 vaccine. To add increased depth and understanding to survey responses, qualitative data were collected using 1:1 semi-structured interviews and allowed for further exploration of vaccine beliefs and intentions.

## Methods

### Context and setting

Data collection occurred in a large urban area in Michigan between mid-April 2021 and March 2022 during the third and fourth waves of COVID-19 [47]. During this time, the vaccine was widely available and recommended to individuals who were 16 years-old or older at pharmacies, local health departments, and nontraditional sites, such as mobile clinics. The vaccine was being heavily promoted during this time, peaking at 3.5 billion ad impressions in May 2021 [48]. To further encourage vaccination, many states in the United States, including Michigan, offered incentives such as free college tuition or entry into sweepstakes, while universities and workplaces were starting to implement vaccine mandate [47]. By the fall of 2021, workplaces and schools were transitioning from remote or hybrid to in-person, and in November 2021, the CDC recommended the primary series of the COVID-19 vaccine for everyone aged 5 years or older, and a booster dose for everyone aged 16 years or older [49–51].

### Design

This parallel convergent mixed-methods study assessed the association between vaccine intentions and asthma control, and the secondary risk theory framework was used to analyze vaccine intentions in Black American emerging adults with asthma. This study consisted of Qualtrics-delivered, investigator-developed surveys, which were analyzed using descriptive statistical methods, and semi-structured interviews, which were analyzed using thematic analysis.

### Participant recruitment, enrollment, and consent

Participants for the current study were selected from the Detroit Young Adult Asthma Project (DYAAP) parent study, which was a randomized controlled trial to improve asthma medication adherence in Black American emerging adults [52]. Only participants who were at least one-month past completion of the intervention stage of the parent study and who had indicated an interest in future studies were contacted. Participants for the COVID-19 sub-study were contacted and enrolled on a rolling basis between April 2021 and March 2022. Sixty participants were eligible and agreed to participate.

Eligibility criteria for the COVID-19 sub-study included: (1) moderate to severe persistent asthma requiring daily controller medications, (2) self-identification as Black American, (3) aged 18–29, (4) English speaking, (5) living within 30 miles of the urban center, (4) access to a cellular phone, and (5) at least one-month past completion of the DYAAP intervention period. No exclusions were made due to comorbid mental health conditions, except thought disorder, suicidality, or intellectual disability. Youth with other serious chronic health conditions were excluded. Enrollment in the COVID-19 sub-study did not impact enrollment in or progression through the DYAAP parent study.

Twenty-five of the 60 participants who were selected for the COVID-19 sub-study were randomly selected by Qualtrics to participate in an additional 1:1 semi-structured interview conducted via Zoom by a trained nursing research assistant.

Participants were compensated with Amazon electronic gift cards after each stage of completion. Participants received a $45 gift card after completing the first questionnaire, a $50 gift card after completing the interview, and a $55 gift card after completing the 3-month follow-up questionnaire.

### Ethical considerations

This study was approved by the university’s Institutional Review Board and informed consent was obtained from all participants prior to data collection. Participants were emailed a copy of the informed consent form that explained the purpose of the study and its associated risks and benefits. Then, a trained research assistant met with participants via Zoom or a telephone call to review the consent form and obtain electronic consent using REDCap software. Informed consent included consent for both survey and interview participation.

### Quantitative data collection and measures

This study includes the results from the baseline Qualtrics questionnaires that participants completed immediately after enrollment. During enrollment, participants indicated whether they would prefer to receive Qualtrics links through email or text, and after completing informed consent, the Qualtrics questionnaires were sent via their preferred method of communication. Links expired 3 days after generation. The last screen of the first Qualtrics questionnaires informed the participant of whether they were randomized to complete an interview. Interviews were conducted within 15 days of completing the surveys.

### Measures

#### Demographics and access to asthma care

Demographic information and a question about access to asthma care were included in this study as they may contribute to participants’ perceptions of risk from COVID-19 and the vaccine. Participants completed an investigator-developed questionnaire to assess both baseline demographic characteristics, such as enrollment in school, employment status, and status as an essential worker, as well as a yes/ no question to assess whether they had trouble accessing asthma care during the pandemic. Sex and age were collected during the parent study and updated during enrollment in the sub-study.

#### Asthma control

To assess the effect of asthma control on vaccine intentions, the Asthma Control Test (ACT) was used [53]. The ACT contains five items, and each item is ranked on a 5-point Likert scale and then summed. Scores range from 5 (poor control) to 25 (complete control) with scores *≥* 20 indicating well-controlled asthma [54]. The ACT has been shown to be valid and have reliability of 0.77 or greater in previous studies [54, 55].

#### Previous experience with COVID-19 and risk beliefs

Four investigator-developed questions were asked to assess participants’ perceived vulnerability from COVID-19. Two yes/ no questions assessed whether the participant had ever tested positive or if they knew anyone who had tested positive for COVID-19. The other two questions asked participants to assess their chance of contracting COVID-19 in the next 3 months and their chance of dying if they contracted COVID-19. These items were ranked on a 4-point scale as follows: 1 (no chance), 2 (low chance), 3 (medium chance), and 4 (very high chance).

#### Vaccine intention

To explore vaccine intentions, participants answered three Likert-style questions that assessed their likelihood of receiving the COVID-19 vaccine, waiting to receive the COVID-19 vaccine, and discussing the COVID-19 vaccine with their healthcare provider. Each item was ranked on a 5-point scale with anchors of 1 (highly unlikely) to 5 (highly likely). Likert scales are commonly used in social science research and measure qualitative data in an ordinal manner [56]. Previous research has shown them to have validity and reliability greater than 0.80 [57].

#### Factors affecting COVID-19 vaccine intention

To assess which factors were most important to participants when choosing whether to receive the vaccine, they were asked to complete an investigator-developed questionnaire to rank the following 10 factors from the most important [1] to the least important [10] in deciding whether to receive the COVID-19 vaccine: safety, efficacy, immediate side effects, long-term side effects, cost, how far they had to travel, how long they had to wait, how bad the COVID-19 outbreak is, whether it is recommended for people with asthma, and how many others have received the vaccine. For ease of analysis and interpretation, the data were reverse-coded prior to statistical analyses so that higher numbers correspond to increased importance.

### Statistical analysis

Statistical analyses were run using IBM SPSS version 29. Descriptive statistics were used to examine participant demographics, risk beliefs, vaccine intentions, asthma control, access to asthma care, and factors affecting vaccine intention for both the survey and interview data. We used frequencies and percentages for categorical variables and means and standard deviations for continuous variables (Table 1). Using the survey data, we ran Spearman rho correlations between vaccine intention variables, age, ACT score, and the ranked factors affecting vaccine intention.

We created dichotomous variables for: high likelihood of dying if contract COVID-19 and high likelihood to receive the vaccine, discuss it with their provider, or wait to receive it. Chi Square tests were then run to test whether gender, employment status, school enrollment, availability of asthma care, employment status, prior infection with COVID-19, knowing someone who tested positive for COVID-19, or having the belief that they were very likely to die from COVID-19 were associated with a high likelihood to receive the COVID-19 vaccine, discuss it with their healthcare providers, or wait to receive it. When a cell contained an expected count of less than 5, we used the Fisher probability instead.

### Qualitative methods

Initially, Qualtrics was set to randomly select 25 participants to participate in an additional interview and receive a message at the end of their first Qualtrics questionnaire that they had been chosen. Research assistants contacted the selected participants to schedule a private 1:1 semi-structured interview. One participant had trouble accessing the Zoom platform, however, and could not be reached to reschedule. Therefore, another participant was randomly selected.

The interviews were conducted by a trained nursing research assistant (AH) who was also a registered nurse practitioner, female, and unknown to the participants. The nursing research assistant had received didactic training in qualitative research methods followed by practical training which included conducting practice interviews with senior research staff who had extensive experience conducting qualitative interviews. Interviews were conducted via Zoom. Participants accessed the interview on their choice of device (e.g., mobile device, computer, tablet), at their choice of location, and at their choice of time. They were able to choose whether to use the video connection (only one participant chose to use video) or audio only. After a brief introduction of her role in the project as a researcher who was interested in the impact of COVID-19 on Black emerging adults with asthma, the interviewer explained the purpose of the interview, reminded participants that they could refuse to answer questions, obtained verbal permission to record the interview, and started the interview process. While the complete interview guide included questions about how the COVID-19 pandemic affected participants’ daily lives and their asthma control, this paper includes only the last question which explored participants’ opinions, intentions, and knowledge about the COVID-19 vaccine (supplemental file 1). Data saturation was achieved as evidenced by no new themes emerging after the first 20 interviews, and interview length ranged between 21 and 52 min. [The COnsolidated criteria for REporting Qualitative Research checklist can be found as supplemental file 2 [58]].

### Qualitative data analysis

Following completion of the last interview, interviews were transcribed and checked for accuracy. Data were analyzed using the thematic analysis approach described by Braun and Clarke [59] and QDA Miner Lite software. Team members (AH and PM) first made notes as they familiarized themselves with the data by reading and rereading the transcribed interviews. Next, codes were generated as AH and PM worked through the entire qualitative dataset looking for patterns across interviews. The next step included identifying these patterns in the data and combining similar and repeated codes into broader themes which were then named and defined. After AH and PM reached consensus upon codes and themes, all interviews were coded by AH. PM coded a 25% subset. The R package tidycomm [60] was used to calculate percent agreement and Holsti’s CR which were both greater than 95%. Percent agreement and Holsti’s CR are appropriate when the data does not require a high degree of interpretation, and there are a large number of codes [61].

## Results

### Quantitative results

Table 1 presents the demographic and clinical characteristics of the Black American emerging adults who completed the surveys and interviews. Fifty-nine participants completed more than 90% of the survey questions and were included in the analysis. (One participant logged in but failed to complete any of the questions despite multiple follow-ups). All survey participants were Black American, 86.4% (*n* = 51) were female, and the mean age was 24.17 ± 2.92 years. The mean asthma control was 17.96 ± 4.35, with 41.1% (*n* = 23) of participants having well-controlled asthma (ACT *≥* 20).

All interview participants were Black American, 88% (*n* = 22) were female, and the mean age was 24.12 ± 2.71 years. The mean asthma control for interview participants was 17.09 ± 3.91 with 30.4% (*n* = 7) of these participants having well-controlled asthma.

Table 1 also includes participants’ beliefs about their risk of contracting COVID-19 and dying if they contract COVID-19. Ten survey participants believed that they were highly likely to die if they contracted COVID-19; however, only one survey participant believed they were highly likely to contract COVID-19.

**Table 1:** Demographic, clinical, and COVID-19 risk characteristics of Black American emerging adults

	Overall Sample *n*(%)	Interview *n*(%)	Overall Sample ${\rm{(\bar X}}\,{\rm{ \pm }}\,{\rm{SD)}}$	Interview ${\rm{(\bar X}}\,{\rm{ \pm }}\,{\rm{SD)}}$
Sex: Female	51 (86.4)	22 (88%)		
Age in years			24.17 ± 2.92	24.12 ± 2.71
Work Full-Time	26 (44.1%)	14 (56%)		
Work Part-Time	13 (22%)	5 (20%)		
Unemployed	18 (30.5%)	6 (24%)		
Essential Worker (n_sam_=39, n_int_=19)	21 (53.8%)	11(57.9%)		
Enrolled in School	13 (22%)	4 (16%)		
ACT (n_sam_=56, n_int_=23)			17.96 ± 4.35	17.09 ± 3.91
ACT *≥* 20 (n_sam_=56, n_int_=23)	23 (41.1%)	7 (30.4%)		
Ever Positive for COVID-19 (n_sam_=48, n_int_=20)	13 (27.1%)	3 (15%)		
Known Someone With COVID-19	39 (66.1%)	17 (68%)		

(n_sam_=survey sample size which was 59 unless otherwise stated, n_int_=interview sample size which was 25 unless otherwise stated)

Participants reported that they would be highly unlikely to receive the COVID-19 vaccine. Fourteen (24.1%) survey participants reported that they would be highly likely to wait, and 24 (40.7%) survey participants reported that they would be highly likely to discuss the vaccine with their primary care provider. Table 2 includes the full set of results for vaccine intentions.

**Table 2:** Vaccine intention frequencies

	Highly Unlikely	Unlikely	Neutral	Likely	Highly Likely
Receive the Vaccine (*n* = 59)	19 (32.2%)	2 (3.4%)	6 (10.2%)	2 (3.4%)	30 (50.8%)
**Receive the Vaccine (interview, ** ***n*** ** = 25)**	**8 (32%)**	**1 (4%)**	**2 (8%)**	**0**	**14 (56%)**
Likely to Wait (*n* = 58)	28 (48.3%)	2 (3.4%)	9 (15.5%)	5 (8.6%)	14 (24.1%)
**Likely to Wait (interview, ** ***n*** ** = 25)**	**10 (40%)**	**1 (4%)**	**3 (12%)**	**2 (8%)**	**9 (36%)**
Likely to Discuss with PCP (*n* = 59)	20 (33.9%)	2 (3.4%)	6 (10.2%)	7 (11.9%)	24 (40.7%)
**Likely to Discuss with PCP (interview, ** ***n*** ** = 25)**	**10 (40%)**	**0**	**4 (16%)**	**2 (8%)**	**9 (36%)**

### Factors affecting COVID-19 vaccination

Safety was chosen by 91.2% participants as one of the three most important factors when deciding to receive the COVID-19 vaccine. Efficacy was chosen as one of the three most important factors by 54.4% participants followed by immediate side effects which was chosen by 49.1% of participants. The full results are in Table 3.

**Table 3:** Number of participants ranking factor as one of the three most important factors affecting their decision to receive the COVID-19 vaccine

Factor	Overall (*n* = 57)	Interview (*n* = 24)
Safety	52 (91.2%)	23 (95.8%)
Efficacy	31 (54.4%)	14 (58.3%)
Immediate Side Effects	28 (49.1%)	12 (50%)
Long-term Side Effects	19 (33.3%)	5 (20.8%)
Cost	3 (5.3%)	2 (8.3)
Distance to Travel	4 (7%)	1 (4.2%)
How Long to Wait	2 (3.5%)	2 (8.3%)
How Bad is Outbreak	7 (12.3%)	2 (8.3%)
Recommended for Asthma	21 (36.8%)	10 (41.7%)
How Many Others Have Received It	4 (7%)	1 (4.2%)

### Bivariate correlations

Bivariate correlations using Spearman’s correlation coefficients were run between vaccine intentions, age, perceived risks from COVID-19, and factors affecting vaccination. (The full results are shown in supplemental file 3). Intention to receive the COVID-19 vaccine was directly correlated to intention to discuss the vaccine with their healthcare provider (ρ = 0.491, *p* < 0.001) and inversely correlated to the importance ranking for immediate side effects (ρ= -0.261, *p* = 0.050) and long-term side effects (ρ= -0.280, *p* = 0.035). Intention to discuss the COVID-19 vaccine with their healthcare provider was directly correlated to asthma control (ρ = 0.339, *p* = 0.011), but inversely correlated with the importance ranking for immediate side effects (ρ= -0.317, *p* = 0.016) and the perceived risk of dying if they contracted COVID-19 (ρ= -0.270, *p* = 0.038). Intention to wait was not correlated with any of the variables tested.

### Chi Square analyses

Being highly likely to receive the vaccine was significantly related to being unemployed (χ^2^ = 12.11, *p* < 0.001), working full-time (χ^2^ = 3.93, *p* = 0.047), and the belief that they were highly likely to die if they contracted COVID-19 (χ^2^ = 8.04, *p* = 0.005). Being highly likely to discuss the vaccine with their healthcare providers was only significantly related to the belief that they were highly likely to die if they contracted COVID-19 (χ^2^ = 4.696, *p* = 0.030). Being highly likely to wait was only significant with testing positive for COVID-19 (Fisher’s exact, *p* = 0.047). None of the participants who tested positive for COVID-19 stated that they were highly likely to wait to receive the vaccine. Full results are in Table 4.

**Table 4:** Chi Square analyses between categorical variables and high vaccine intentions

	Highly Likely Receive the Vaccine (*n* = 59)	Highly Likely to Discuss It with Healthcare Provider (*n* = 59)	Highly Likely to Wait to Receive the Vaccine (*n* = 58)
Gender (*n* = 59)	χ^2^(1, *N* = 59) = 0.50, *p* = 0.48	χ^2^(1, *N* = 59) = 0.94, *p* = 0.33	χ^2^(1, *N* = 58) = 0.69, *p* = 0.41
Enrolled in School (*n* = 59)	χ^2^(1, *N* = 59) = 0.76, *p* = 0.38	χ^2^(1, *N* = 59) = 1.20, *p* = 0.274	χ^2^(1, *N* = 58) = 0.006, *p* = 0.94
Unemployed (*n* = 59)	χ^2^(1, *N* = 59) = 12.11, *p* < 0.001	χ^2^(1, *N* = 59) = 0.15, *p* = 0.70	χ^2^(1, *N* = 58) = 1.64, *p* = 0.20
Employed Full-Time (*n* = 59)	χ^2^(1, *N* = 59) = 3.93, *p* = 0.047	χ^2^(1, *N* = 59) = 0.10, *p* = 0.76	χ^2^(1, *N* = 58) = 0.62, *p* = 0.43
Essential Worker (*n* = 39)	χ^2^(1, *N* = 39) = 0.10, *p* = 0.76	χ^2^(1, *N* = 39) = 0.96, *p* = 0.33	χ^2^(1, *N* = 39) = 0.06, *p* = 0.81
Tested Positive (*n* = 48)	χ^2^(1, *N* = 48) = 0.001, *p* = 0.98	χ^2^(1, *N* = 48) = 1.10, *p* = 0.30	Fisher’s exact, *p* = 0.047
Known Someone Who Had COVID (*n* = 59)	χ^2^(1, *N* = 59) = 3.04, *p* = 0.081	χ^2^(1, *N* = 59) = 0.23, *p* = 0.63	χ^2^(1, *N* = 58) = 0.07, *p* = 0.79
Healthcare Access (*n* = 59)	χ^2^(1, *N* = 59) = 2.12, *p* = 0.15	χ^2^(1, *N* = 59) = 0.09, *p* = 0.76	χ^2^(1, *N* = 58) = 0.57, *p* = 0.45
Highly Likely to Die if Contract COVID (*n* = 59)	χ^2^(1, *N* = 59) = 8.04, *p* = 0.005	χ^2^(1, *N* = 59) = 4.70, *p* = 0.03	χ^2^(1, *N* = 58) = 0.99, *p* = 0.32

### Qualitative results

Themes and codes were applied to the interviews, and then the interviews were stratified according to vaccine intention into three groups for analysis: 13 participants who had already received the vaccine were assigned to the group “Received”, four participants who were either planning to receive the vaccine or considering it were assigned to the group “Considering”, and eight participants who were not planning to receive the vaccine were assigned to the group “Not Likely”. Five overarching themes emerged from the interviews: beliefs about the safety of the vaccine, beliefs about the efficacy of the vaccine, personal factors affecting the decision to receive the vaccine, societal factors affecting the decision to receive the vaccine, and general knowledge and information seeking about the COVID-19 vaccine. Table 5 includes quotations which are representative of the selected themes as well as the participants’ vaccine intentions.

**Table 5:** Representative quotes supporting themes from qualitative research

Theme	Vaccine Intention	Representative Quotations
Safety: Speed of development as source of vaccine hesitancy	Received	“I was definitely kind of like, oh wow, that was fast, and I was kind of a little nervous to get it because of that”
Safety: Concern about immediate side effects	Not Likely	“um, like, heart problems, breathing problems, and fevers, headaches, and you get the coronavirus, and all of that”
Efficacy: Vaccine improving anxiety about coronavirus	Received	“I am still pretty worried [about COVID], um not as worried as I was, you know before I got vaccinated”.
Efficacy: Still question efficacy after vaccination	Received	“I don’t know, I don’t know if it’s working or not, and I don’t really want to find, I don’t want to find out either, but I just, I just hope that it is, you know, doing something that it, that it should”
Efficacy: Thoughts about variants and need for boosters	Not Likely	“It’s like any other [vaccine], like the flu vaccine, it’s not gonna stop you from getting the flu, it’s just decreasing your chances. And then, you know with the new strains, it’s kind of making it ineffective, like it’s already obsolete”
Personal Factors: Protect self and others	Considering	Um, just because um, my mom is older and I have really bad asthma and um, I’ve seen a lot of people pass away because of COVID and I don’t want to be one of them.”
Personal Factors: Protect self and others	Received	I feel like it’s better to have it [the vaccine] and be a little sick rather than to not have it, and possibly be extremely sick or die from it [COVID] or passing it on to somebody who can die from it. So it’s kind of one of those things. You have to weigh the lesser evils and choose what you think is best.”
Personal Factors: Protecting self other ways	Not Likely	“if I am taking the right precautions, maybe it’s not needed as of right now”
Information Gathering: Research lowered hesitancy	Received	“Um, so, when I first heard about it, I think like a lot of other people, I was definitely kind of like, oh wow, that was fast, and I was kind of a little nervous to get it because of that. I did my research which, I discovered that we had SARS back in the day. They had been doing research with a vaccine regarding that and they used that as a blueprint for um, the groundwork for the vaccines right now. So because of that right there, kind of made me feel a lot more comfortable”
	Considering	“At first, I wasn’t sure about it, but once I did my research, I realized it’s a good idea to get it to protect me and others around me.”
Information Gathering: Information from friends, family, news about side effects impacting vaccine intention	Not Likely	“There was a couple of people who actually died from getting the vaccine, now granted, there may have been some underlying issues, but of course, the media, saying well, they were young, they were healthy, and they got the vaccine, and then something went wrong.”
Information Gathering: Thoughts on social media	Received	“You got to be careful about social media in the sense that a lot of people just post BS on social media all of the time”
Societal Factors Affecting Decision to Vaccinate: Availability	Considering	“You can go to the Rite-Aid or anywhere to receive it.”
Societal Factors Affecting Decision to Vaccinate: Incentives	Not Likely	“they are providing weird and gross incentives to get people to come. Those are, like I said, things that I’ve specifically read that go against the medical community’s law of ethics”
Societal Factors Affecting Decision to Vaccinate : Protected because of unemployment, businesses closed, and/ or not going out as much	Not Likely	“considering that you know if I was out and about and active, maybe I would get it, but considering that I’m home most of the time anyways, I feel like I can wait on that”
Societal Factors Affecting Decision to Vaccinate : Reopening society	Not Likely	“I think it’s so that we can get the economy moving again in a way that is going to be positive for these different businesses out there. I feel like they want to, you know, stop the unemployment benefits as soon as they can because that’s putting a damper on the economy…so I feel like a lot of the motivation behind a vaccine is purely monetary… and that makes me a lot less likely to participate in receiving the vaccine”

#### Beliefs about vaccine safety

All participants discussed their beliefs about the safety of the COVID-19 vaccine, and participants in all three groups were concerned about the speed with which the vaccine was developed. Beliefs about the immediate side effects of the vaccine varied, however, between those who had received the vaccine and the other two groups. Participants who received the vaccine were more likely to discuss the immediate side effects that they had as minimal or positive. For example, one participant stated, “it [immediate side effects] did resemble a lot of the symptoms that I experienced when I had COVID, which is good, because it is like my body, you know was fighting off COVID”. Participants in the other two groups were more likely to discuss both known and unproven serious adverse effects, including getting COVID from the vaccine and death.

#### Beliefs about vaccine efficacy

Many participants discussed their beliefs about the efficacy of the COVID-19 vaccines. Participants in all three groups discussed how the vaccine does not stop them from contracting COVID-19; however, it does decrease the likelihood of serious outcomes if they contract COVID-19. Being vaccinated decreased worries about the coronavirus for some participants while others who had been vaccinated still expressed doubt. In later interviews, participants in all three groups were questioning the efficacy of the vaccine related to emerging variants and the recommendation for booster doses.

#### Personal factors affecting the decision to vaccinate

Participants who received the vaccine and who were considering it discussed receiving the vaccine to protect themselves and others, such as their parents, grandparents, people they worked with, and their children. Having asthma was also mentioned as a motivator for receiving the vaccine. Several participants who were not planning to receive the vaccine stated that they did not feel that it was necessary because they were protecting themselves in other ways. While several of the participants who received the vaccine believed that others should receive it, too, other participants who received the vaccine, those who were hesitant, and those who were considering it discussed that getting the vaccine was a personal choice.

#### Information gathering

There were participants in all three groups who discussed how information gathering changed their vaccine intention. Participants who had received the vaccine or were considering it discussed how doing their own research increased their confidence in the vaccine; however, one of the participants who was not likely to receive the vaccine discussed how they were initially excited about the vaccine, but then changed their intention as they felt that the vaccine was being promoted for non-public health reasons. Most of the participants who were considering the vaccine or who had received it identified the CDC and the State of Michigan’s Department of Health and Human Services (MDHHS) websites as trusted sources. Other trusted sources across groups included college professors, family, and friends. Participants who were not likely to receive the vaccine discussed information that they received from family, friends, or the news about the vaccine’s more serious side effects as a deterrent to vaccination. Across groups, participants discussed how they felt hesitant about information found on social media.

#### Societal factors affecting the decision to vaccinate

Three societal factors emerged that affected vaccine intention: availability of the vaccine, vaccine incentives, and social restrictions. Several participants reported receiving the vaccine when it was offered because they were worried that they would not be able to get it if they waited, but during later interviews, participants stated that availability was no longer an issue, that the vaccine was widely available, and they knew how to get it if they wanted it. As vaccines became widely available, incentive programs emerged to increase vaccination. The participants who mentioned the incentives noted that the incentives contributed to their mistrust.

Participants also discussed how restrictions on society affected their decision about whether to receive the vaccine, and how their decision may change if society opened back up. Several participants who were not planning to receive the vaccine stated that they were not planning to receive the vaccine at that time because they were not employed, or they were not going out as much. One interesting perspective arose in which a participant expressed concern that the vaccine was not being promoted for public health reasons, but to give people confidence to reopen society. This participant had voiced a similar concern earlier in the interview that, “we’re rushing to get back to something that wasn’t really working for us anyways”.

## Discussion

Black American emerging adults are at an increased risk for poor asthma outcomes which include increased ED use and death [62, 63], and vaccines, including the COVID-19 vaccine, are an important part of asthma self-management [64]. Yet, little is known about vaccine intentions in Black American emerging adults with asthma. Our study showed that even at the height of a global COVID-19 pandemic, despite aggressive marketing and official recommendations from official sources, only 54% of the Black American emerging adults with asthma in our sample were either likely or highly likely to receive the primary series of the COVID-19 vaccine. These results are much lower than those found in a study by Stoner and colleagues where more than 80% of the Black American emerging adults had received the primary series [43]. Our results were similar, however, to the results of a 2022 meta-analysis of the general population in which vaccine acceptance was 56% among emerging adults aged 18–29 and 44% among Black Americans [33]. COVID-19 vaccine acceptance in our study was also lower than the 50–67% vaccination rate that has been proposed to confer herd immunity [65].

Consistent with secondary risk theory and existing literature [26–32], over 90% of the participants chose safety as one of the three most important factors they considered when deciding whether to receive the COVID-19 vaccine. Not surprisingly, likelihood to receive the COVID-19 vaccine was inversely correlated with concern about immediate and long-term side effects. Interview data suggested that while safety was important to most Black emerging adults with asthma, perceptions of the COVID-19 vaccine’s safety differed based upon vaccine intention. For example, Black American emerging adults who received the vaccine or were considering it were more likely to discuss side effects as minimal or positive, while those who were not planning to receive it discussed both real and hypothetical serious events that have been associated with receiving the vaccine, including myocarditis, neurological concerns, contracting COVID from the vaccine, and death. Regardless of vaccine intention, the speed with which the vaccines were developed contributed to safety concerns; however, consistent with previous literature [30], several participants who received the vaccine discussed how increasing their scientific knowledge about the vaccines’ development alleviated their concerns.

Over 50% of Black American emerging adults with asthma chose efficacy as one of the top three concerns that would impact their decision to receive the COVID-19 vaccine, which is also consistent with secondary risk theory and previous literature [32]. Participants who received the vaccine discussed during the interviews that they felt less anxious about COVID-19 and returning to pre-pandemic activities; however, regardless of vaccine intention, participants questioned the efficacy of the vaccines as new variants emerged and booster doses were recommended with one vaccine-hesitant participant stating that the vaccines were already obsolete.

Only 17% of unemployed participants were highly likely to receive the primary series of the COVID-19 vaccine compared to 65% of participants who were not unemployed. During the interviews, several participants stated that they were not receiving the vaccine due to societal factors, such as businesses being closed or being unemployed. Thus, employment status may have served as a proxy for participants’ sense of vulnerability from COVID-19. Conversely, participants who believed that they had a high likelihood of dying if they contracted COVID-19 were less likely to state that they were highly likely to receive the vaccine or discuss it with their provider. One explanation for this finding may be that only one participant in the sample felt that they were highly likely to contract COVID-19 in the next three months. Indeed, none of the 13 participants who had tested positive for COVID-19 indicated that they were highly likely to wait to receive the vaccine.

Intention to receive the COVID-19 vaccine was correlated with intention to discuss the vaccine with their healthcare providers, but just over half of our participants stated that they were likely or highly likely to discuss the COVID-19 vaccine with their healthcare provider. Importantly, improved asthma control was correlated with intention to discuss the COVID-19 vaccine with their healthcare providers, but it was not significantly correlated with intention to vaccinate. Communication with healthcare providers is an important part of asthma self-management [66], and our survey suggested that there were not significant relationships between whether participants were able to receive care from their asthma healthcare provider and their intention to discuss the vaccines with their provider, receive it, or their asthma control. This finding suggests that Black American emerging adult patients, especially those with lower asthma control, who are still receiving asthma-related care may benefit from provider-initiated conversations about COVID-19 vaccines. Indeed, when healthcare providers recommended the COVID-19 vaccine to other populations, even to patients who are vaccine-hesitant, patients were more likely to be vaccinated [67].

Contrary to secondary risk theory, participants who believed that they were very likely to die if they contracted COVID-19 were less likely to discuss the COVID-19 vaccine with their healthcare provider. One explanation for this finding may be that only one survey respondent believed they were highly likely to contract COVID-19, and 50 of our 59 participants felt that they had a small or no chance of contracting COVID-19. Participants who knew someone who tested positive may have felt more vulnerable to COVID-19, as this factor correlated with an increased, although not significant, relationship with being more likely to receive the COVID-19 vaccine. Societal factors may have contributed to participants feeling less vulnerable to the threat from COVID-19 than to the threat from the vaccine itself. In both adjusted and unadjusted models, participants who were unemployed were less likely to be highly likely to receive the COVID-19 vaccine. Several interview participants stated that they were not receiving the vaccine either because they were unemployed or because businesses were not yet open, and they were protecting themselves by staying home and social distancing. Two of these participants stated that if businesses reopened or they returned to work, they would receive the vaccine.

## Conclusion

Black American emerging adults with asthma were less likely than the general population to report intention to receive the COVID-19 vaccine and were most concerned about safety, efficacy, and side effects. We did not find a relationship between the ability to attain asthma-related care and intention to discuss the COVID-19 vaccine with their healthcare provider; however, intention to discuss the COVID-19 vaccine with their healthcare provider was correlated with intention to vaccinate and asthma control. These findings would suggest that Black emerging adult patients with poorer asthma control may benefit from provider-initiated discussions about vaccines. Because societal conditions and restrictions also contributed to vaccine hesitancy, ongoing discussions about vaccine intentions between healthcare providers and Black American emerging adults with asthma may improve vaccine acceptance.

## Limitations

There were several limitations within our study. First, we did not ask our participants in the surveys if they had received the COVID-19 vaccine, although we did ask them during the interview. It is possible that participants who had already received the COVID-19 vaccine may have been confused by the question. Indeed, one participant stated during the interview that they had received the vaccine but chose that they were highly unlikely to receive it on the survey and typed in a statement on the survey that they had received the vaccine (their survey response was changed to reflect the typed-in statement). Thirteen of the 14 interview participants, however, who chose that they were highly likely to receive the vaccine on the surveys indicated that they had already received it during their interviews.

Several other discrepancies emerged between the survey responses and the interview responses. Two participants who stated that they were planning to receive the vaccine during the interview answered that they were highly unlikely to receive the vaccine in the survey. One possible explanation may be that receiving the vaccine was seen as socially desirable in the context of speaking with health researchers during the interviews. When answering the questions anonymously on the surveys, participants may have felt more comfortable being honest. Wolter and colleagues found similar social desirability in that participants’ self-report of COVID-19 vaccination in Germany was higher than the actual vaccination rate attained from physicians and official sources [68].

Second, our data were collected on a relatively small convenience sample in an urban area and may not be generalizable to other populations, including Black American emerging adults with asthma in rural or suburban areas. Third, our data were self-reported and cross-sectional in nature. The data were collected over the timeframe from April 2021 through February 2022 when there were rapid changes in society. By September 2021, booster doses were being recommended in addition to the primary series, society was gradually opening back up, and workplaces and universities were mandating vaccines. These changes may have affected the vaccine attitudes and intentions of Black American emerging adults with asthma, as we were starting to see in later interviews. For example, several interviewees stated that they would be more likely to receive the vaccine if society opened back up or they returned to work; therefore, if they had been surveyed and interviewed later, their responses may have changed.

Despite these limitations, this study adds to the limited asthma self-management literature in Black American emerging adults. To our knowledge, this the first study which explores the relationship between asthma control in Black American emerging adults and the intention to receive vaccines recommended for individuals with asthma. Additionally, it is the first study which explores the relationship between asthma control in Black American emerging adults and their intention to discuss recommended vaccines with their healthcare provider.

### Electronic supplementary material

Below is the link to the electronic supplementary material.

Supplementary Material 1

Supplementary Material 2

Supplementary Material 3

## Data Availability

The quantitative dataset used and/ or analyzed during the current study is available from the corresponding author upon reasonable request; however, the qualitative dataset used and/ or analyzed during the current study is not available to protect the anonymity of the research participants.
