# Supplementary material for: COVID-19 vaccine intentions and attitudes in Black American emerging adults with asthma
Source: BMC Public Health. 2024 May 20;24:1356. doi: 10.1186/s12889-024-18843-w (PMC11106870; doi:10.1186/s12889-024-18843-w)
Supplement: Supplementary file 1 — Supplementary Material 1 [file 12889_2024_18843_MOESM1_ESM.docx]

Supplemental File 1: Vaccine Questions from Interview Guide

Vaccine Question:

Now, I’d like to talk about the vaccine for COVID-19. Could you tell me what you know about the vaccine?

Probe 1: Have you received the vaccine? If YES, prompt: Could you tell me how that was for you? Did you experience side effects? If NO: Will you get the vaccine if it is available to you? Why or why not? If only one/partial dose (if 2 dose vaccine): Will you get the second dose? Why or why not?

Probe 2: Do you know how and where to sign up for the vaccine?

Probe 3: What is your opinion about the vaccine at this point? Has that view changed at all?

Probe 4: Do you have any concerns about the COVID vaccine? (Follow-ups, if not answered: Are you concerned about effectiveness? If have not received vaccine: Are you concerned about side effects? Do you have concerns about being able to get the vaccine if you want it?)
